# Sexual functioning is not, but psychological burden is predictive for receiving help in pelvic physical therapy practice: A cross-sectional study

**DOI:** 10.12688/openreseurope.16138.2

**Published:** 2024-05-20

**Authors:** Alma Brand, Wim Waterink, Jacques van Lankveld

**Affiliations:** 1 Faculty of Psychology, Open University of The Netherlands, Heerlen, Limburg, 6419 AT, The Netherlands

**Keywords:** Pelvic Floor Complaints, Sexual Functioning, Psychological Burden, Pelvic Physical Therapy, Predictive value

## Abstract

**Background:**

Pelvic floor complaints are common among women and often accompanied by sexual dysfunction and psychological burden. They are also associated with pregnancy and childbirth. However, not all women with these complaints receive help in pelvic physical therapy practice. This study explored if pregnancy, parity, pelvic floor complaints, sexual functioning, and psychological burden are predictive of receiving help in pelvic physical therapy practice.

**Methods:**

In a cross-sectional exploratory design, women completed an online survey about pelvic floor complaints, sexual function, and psychological burden. Binary logistic analysis was used to analyze the predictive value of the above-mentioned factors.

**Results:**

Data from 542 participants were analyzed. Pregnancy and parity, PFC severity, psychological burden, and the interaction between pelvic floor complaints and psychological burden were significant predictors of receiving help. Against expectations, sexual functioning was not predictive of receiving help.

**Conclusions:**

Women’s psychological burden is an important factor in determining if or when women receive help in PPT practice. More research is needed to unravel the role of sexual functioning in the context of pelvic floor complaints and women’s psychological burden. More insight into this area of expertise could possibly improve and enhance pelvic health care for women with pelvic floor complaints.

## Plain language summary

Women with pelvic floor complaints may also experience sexual dysfunction and psychological burdens. Their complaints can be related to pregnancy and childbirth. However, not all women with pelvic floor complaints receive help in pelvic physical therapy practice. This study explored if pregnancy, childbirth, pelvic floor complaint severity, sexual functioning, and psychological burden predicted women’s help-seeking behavior in pelvic physical therapy practice. For that purpose, women were invited to complete an online survey; data from 542 participants were analyzed. Outcomes revealed that pregnancy, childbirth, and pelvic floor complaint severity predicted help-seeking behavior. Against expectations, sexual functioning did not predict this help-seeking behavior. Psychological burden turned out to be an important predictor. More research is needed to unravel the role of sexual functioning in the context of pelvic floor complaints and women’s psychological burden. Knowing more about these factors may improve and enhance pelvic health in many women.

## Introduction

Many women experience pelvic floor complaints (PFC) such as micturition and defecation problems, urinary and fecal incontinence, pelvic organ prolapses, pelvic pain, and painful intercourse ^1– 8^. These complaints can be symptomatic of pelvic floor dysfunction (PFD), the inability to relax or contract the pelvic floor muscles adequately ^9^, which can be treated in pelvic physical therapy (PPT) practice ^10, 11^. In Dutch women aged between 18 and 45 years, the prevalence of urinary incontinence is estimated at 25–50%, with higher rates during pregnancy and at older ages ^12– 16^. Micturition problems, such as cystitis and urgency are found in approximately 10% of Dutch women ^12– 16^. The prevalence of cystitis tends to increase during pregnancy, and after coitus when condoms or coils with sperm-killing lubricants are used ^12– 16^. The prevalence of defecation problems in women varies between 5–35% ^17, 18^, and the prevalence of pelvic organ prolapse is more than 40% in women over 40 years of age ^19^. Pelvic pain is reported by 14–24% of women in the Netherlands, with an increase to more than 60% during pregnancy and more than 30% after childbirth ^13, 20– 22^. Recurrent genital pain and painful intercourse are reported by 65% of women after childbirth, decreasing to less than 30% at one year postpartum. Approximately 21% of women experience at least one painful sexual encounter in their life ^13, 22– 24^.

PFCs are often associated with sexual health problems or sexual dysfunction ^1– 8^. Sexual health is defined as a state of physical, emotional, spiritual, and social well-being about sexuality, emphasizing sexual pleasure ^25^. Sexual dysfunction is described as experiencing problems with desire, arousal, orgasm, or pain during sexual activity that impair satisfactory experiences in combination with distress ^26^. In the Netherlands, 21% of women suffer from sexual dysfunction ^27, 28^. Decreased sexual desire is present in 7%, lubrication problems in 6%, painful intercourse in 5%, and orgasm problems in 4% of these women ^28^. Sexual dysfunction was found to induce feelings of distress, such as fear, anxiety, avoidance, insecurity, performance dissatisfaction, shame, guilt, and decreased sexual pleasure ^29^. This distress increases even further in the presence of PFC ^30– 34^. Women with PFC also experience various types of distress. Loss of control over pelvic floor muscle function often triggers insecurity ^35^. Incontinence is frequently linked to embarrassment ^3, 5, 36^. Pelvic pain tends to induce distress, such as a feeling of loss of control, and being dependent on others for help ^37– 39^. PFC, the associated sexual health problems, and distress appear to vary with pregnancy and childbirth ^3, 12– 16, 20– 24^. In this study, women’s distress with PFC will be further referred to as a psychological burden.

Not all women with PFC experience sexual health problems and a high psychological burden, and not all women receive help ^3, 40^. When women receive help, they make a conscious decision to do so in the hope of improving their pelvic of sexual health ^41^. However, other women with PFC do not receive help for various reasons, including the appraisal of their complaints as not severe enough, as part of the aging process, or as insoluble. These women might try to adapt their behavior to deal with complaints by themselves, or to accept their complaints ^12, 40^. Because women with PFC often receive help from pelvic physical therapists, who are trained to treat pelvic floor dysfunction and pelvic floor-related sexual dysfunction, the healthcare setting in which help is sought in this study, is PPT practice.

This study explores the predictive value of PFC severity, women’s level of sexual functioning, and their psychological burden for seeking help in PPT practice. This is investigated in pregnant, parous, and nulliparous women. Knowing if these factors predict if these groups of women receive help in PPT practice, might offer an opportunity to better understand the help they need and to—ultimately—improve pelvic health in young adult women. Information could then be proactively given to young adult pregnant, parous, and nulliparous women, and adequate care could be initiated early. Therefore, the main research question is: To what extent do PFC severity, sexual functioning, and psychological burden predict if women receive help in PPT practice? It is expected that Pregnancy, Parity, PFC severity, sexual functioning, and psychological burden are predictive of seeking help.

## Methods

### Ethical considerations

The study protocol was approved by the Ethical Review Board of the Open University of the Netherlands on May 29th, 2019/No. U2019/03973/HVM. Participants gave informed written consent to participate and were assured of anonymous use and publication of the data they provided.

### Study design

A cross-sectional study utilizing an online survey was performed. Given the intimate character of the questions that were asked, the survey was completed online, considering that online questionnaires are cheap, easily available, accessible, and convenient. Furthermore, the use of online questionnaires reduces the amount of socially desirable answers, the risk of problems through data loss, and errors from manual entry in the datasheet of participants’ responses ^42, 43^.

### Participants

Pregnant, parous, and nulliparous Dutch women with and without PFC, aged between 18 and 45 years were included in this study. Pregnant women who were expecting their first baby, parous women who had a child younger than two years old, and nulliparous women who had no children and were not pregnant were included. Participants were recruited with help from pelvic and general physical therapists, midwives, general practitioners, and medical specialists, and through social media using targeted invitations and advertisements, websites, E-mail, verbal invitation, and the use of the snowball method through the social networks of participants and recruiters. This was done to avoid nonrandom recruitment as much as possible ^43^. In a later stage of the recruitment period, targeted recruitment was used through a project on the website Hersenonderzoek.nl ( www.hersenonderzoek.nl), a paid service. To avoid bias, pelvic physical therapists were excluded from participation. Self-selection bias may have occurred because participation was voluntary. The fact that the main researcher recruited participants in her own practice, may have caused some response bias. To minimize this type of bias, all the above-mentioned recruitment methods were applied. 

### Study preparation and data collection

The online survey comprised three parts to offer participants the option to answer the questions in stages. The first part of the survey contained demographic and eligibility questions. The presence and type of partner at the time of participation were inventoried with the options: one male partner, one female partner, a dating partner, multiple partners, or no partner. In addition, participants were asked to indicate receiving or not receiving PPT at the time of participation to sort them into two groups of women, respectively, receiving and not receiving help.

In the next two parts of the survey, the severity of seven common types of PFC was inventoried. All questions referred to complaints during the past month. Questions from the Pelvic Floor Distress Index (PFDI) ^44^ were used to identify the presence and severity of five organ-related PFC: *Urinary Incontinence* (UI; items 16 and 17), *Fecal Incontinence* (FI; items 9 and 10), *Micturition Problems* (MP; items 5, 15, and 19), *Defecation Problems* (DP; items 7, 8, and 12), and *Pelvic Organ Prolapse* (POP; item 3). Answering options ‘not present’ and ‘no bother’ indicated the absence (score 0) of a complaint, and the answer options 'a little bother’ (score 1), ‘some bother’ (score 2), and ‘a lot of bother’ (score 3) indicated presence and severity. The severity scores of the organ-related PFC were calculated by dividing the scores on UI and FI by two, and the scores on MP and DP by three to align scores with the POP severity scores. In addition, the presence and severity of *Pelvic Pain* (PP) were questioned using question 5 from the Four-Dimensional Symptom Questionnaire (4DSQ) ^45, 46^ in addition to low back pain, pelvic pain, and coccyx pain were included in the question because these pain complaints are often presented in PPT practice. Answering options ‘not present’ and ‘sometimes’ indicated the absence (score 0) of PP and the answer options ‘regularly’ (score 1), ‘often’ (score 2), and ‘continuously’ (score 3) indicated the presence and the severity of PP. Before asking questions about sexual functioning, participants were asked if they had experienced any sexual violence or abuse (sexual trauma) in their lives. If negative sexual experiences influence women’s level of sexual functioning, it should be included as a factor in the analyses. The PFC *Painful Intercourse* (PI) was questioned, using question 17 of the Female Sexual Functioning Index (FSFI) ^47^ in the third part of the survey. Responses were reverse-scored to align them with the other PFC scores. The answering option ‘never’ indicated the absence (score 0) of PI and the answer options ‘a few times’ and ‘sometimes’ (score 1), ‘mostly’ (score 2), and ‘always’ and ‘did not try having intercourse’ (score 3) indicated the presence and the severity of PI. The maximum PFC severity score was 21. All PFC scores higher than ‘0’ were recoded into ‘1’ indicating their presence to be able to calculate how many participants did not have any PFC, and how many had one or more PFC.

The Pelvic Floor Complaint-related Psychological Burden Inventory (PFC-PBI; 10 items) was used to inventory women’s psychological burden with PFC. The psychological burden scores used in this study are calculated using the ten items that best represent this burden based on the new scale that was developed in prior research ^48^. A score of ‘0’ indicated the absence of psychological burden. Higher scores on the 7-point Likert-type scale indicated higher applicability of the statement’s content and therefore presence and severity of psychological burden. The maximum score on this scale was 60.

In the third part of the survey, women’s level of sexual functioning was assessed using four subscales of the Female Sexual Functioning Index (FSFI) ^47^: desire, arousal, lubrication, and orgasm. Despite critical reviews ^49^, the FSFI is still the most commonly used instrument to measure female sexual functioning. FSFI satisfaction scores were excluded because not all participants had a partner or relationship, and pain scores were excluded because a question from this subscale was used to inventory the PFC painful intercourse. The FSFI scores in the four included subscales were coded and calculated according to their guidelines ^50^. Their sum score represented women’s overall level of sexual functioning, with higher scores representing a higher level of sexual functioning. The maximum score on this scale was 24.

### Data collection procedures

All participants received a link to a secured online data acquisition portal O4U, where they received information about the purpose of the study and other participant information. Participants were required to sign the online informed consent form in the research environment to gain access to the questionnaires. It took the participants approximately 20 minutes to fill out the questionnaires. Participants could quit participation at any time. On completion, they received a debriefing message, thanking them for their participation. Data were collected from November 2021 until March 2023.

### Data analysis

A power analysis in G*Power 3.1 was performed for logistic regression analysis with five predictors ^51^. Two predictors were nominal variables, and three were scale variables. It was decided to estimate the odds ratio at 1.02 based on the variable psychological burden, which had the widest scoring range (0–60) to obtain a meaningful effect with practical significance. The probability to accept or reject the hypothesis was estimated at 0.50 in a two-tailed analysis. Based on preliminary calculations with collected data on women’s psychological burden, a mean value of 15 and a standard deviation of 15 were used in the calculation. In addition, normal distribution, and a power of 0.80 were assumed. A minimal total sample size of 380 participants was needed.

Data analyses were performed using SPSS 28. Data from women who fully completed all three parts of the survey were included, which resulted in the absence of missing data. Descriptive statistics were run to provide general information about the study sample. To explore possible predictive patterns for seeking help, mean scores on the three predictive scale variables PFC severity, sexual functioning, and psychological burden were calculated for women who did and did not receive PPT at the time of participation. To explore the associations between the study variables, correlations were calculated between the sum scores of PFC severity, sexual functioning, and psychological burden for women receiving and not receiving PPT for the total sample, and for pregnant, parous, and nulliparous participants separately. The data were then checked for univariate and multivariate outliers before further analyses ^52^. Multivariate Analyses of Variance (MANOVA) was used to identify group differences in PFC severity, and sexual functioning, and univariate Analysis of Variance [ANOVA] was used to explore between-group differences in psychological burden. Before further analyses, PFC, sexual functioning, and psychological burden scores were standardized to be able to compare predictive outcomes. Binary logistic regression analysis was performed with receiving or not receiving PPT as the to-be-predicted (statistical criterion) variable, indicating membership of a group. Age will be added as a covariate if a significant difference between the two PPT groups is found ^53^. In addition, the interaction terms between predictors were entered into the model.

## Results

A total of 542 participants completed the three parts of the survey. The participants' mean age was 31.22 years, 31.45 years in the sample without PPT, 30.48 years in the PPT sample, 30.92 years in pregnant, 32.10 years in parous, and 30.71 years in nulliparous women. The age difference between the subgroups receiving and not receiving PPT was not significant. The mean gestation age of pregnancy was 25.75 weeks, 24.00 weeks in the sample without PPT, and 27.03 weeks in the PPT sample. Parous women had on average 1.52 children, 1.47 in the sample without PPT, and 1.61 in the PPT sample. In total, 412 participants had a male partner, 11 had a female partner, and 18 had a dating partner. Four participants had multiple partners, and 97 participants did not have a partner. Additionally, 101 participants (18.63%) reported a history of negative sexual experiences. No significant difference was found in the level of sexual functioning between women with and without negative sexual experiences (t=0.331, *p*=0.741). Table 1 shows the included number of participants in each PPT group in pregnant, parous, and nulliparous participants and in the total sample.

**Table 1.** Numbers of participants with and without pelvic floor complaints, and with and without pelvic physical therapy.

		No PPT (N=413)	PPT (N=129)	Total N per subgroup	N
**Pregnant**	No PFCs	0	0	0	** 59**
	PFCs	25	34	59	
**Parous**	No PFCs	9	0	9	**189**
	PFCs	114	66	180	
**Nulliparous**	No PFCs	29	0	29	**294**
	PFCs	236	29	265	
**Total sample**	No PFCs	38	0	38	**542**
	PFCs	375	129	504	

Note: PPT = Pelvic physical therapy, PFCs = Pelvic floor complaints

Table 2 shows the correlations between the key study variables. In the full sample of participants, the correlation coefficients between PFC severity and level of sexual functioning were negative and significant, showing lower sexual functioning with more severe PFC. The correlation coefficients between PFC severity and level of psychological burden were positive and significant, showing a higher psychological burden with more severe PFC. Furthermore, significant negative correlation coefficients were found between the level of sexual functioning and psychological burden, showing a higher psychological burden with lower sexual functioning. This pattern existed in all subgroups of participants but not all correlation coefficients were significant in all subgroups. In pregnant and parous women, receiving and not receiving PPT, the correlation coefficients of sexual functioning and psychological burden were not significant. In nulliparous women who received PPT, no significant correlation coefficients were found between PFC severity and the level of sexual functioning.

**Table 2.** Correlations between pelvic floor complaints, sexual functioning, and pelvic floor complaint-related psychological burden in the full sample, and in pregnant, parous, and nulliparous women receiving and not receiving pelvic physical therapy.

Help		No pelvic physical therapy		Pelvic physical therapy	
		Pelvic floor complaint severity	Sexual functioning	Pelvic floor complaint severity	Sexual functioning
**Total sample**	Sexual functioning	-0.436 ***		-0.355 ***	
	Psychological burden ^a^	0.590 ***	-0.292 ***	0.483 ***	-0.213 *
**Pregnant** ** women**	Sexual functioning	-0.644 ***		-0.509 **	
	Psychological burden ^a^	0.714 ***	-0.351	0.568 ***	-0.203
**Parous women**	Sexual functioning	-0.356 ***		-0.336 **	
	Psychological burden ^a^	0.619 ***	-0.162	0.519 ***	-0.150
**Nulliparous ** **women**	Sexual functioning	-0.459 ***		-0.227	
	Psychological burden ^a^	0.569 ***	-0.345 ***	0.379 *	-0.370 *

Note: * *p*<0.05, ** *p*<0.01, *** *p<*0.001. ^a^ Pelvic Floor Complaint-related Psychological Burden.

Table 3 shows the prevalence of (types of) PFC in the full sample. Mean scores and standard deviations of PFC severity, level of sexual functioning, and psychological burden in women receiving and not receiving PPT are also shown in this table. Before further analyses were performed, data were checked for outliers. Based on univariate outlier analyses of PFC severity scores, data from 10 participants were excluded from further analyses. Subsequently, based on univariate outlier analyses of sexual functioning data from another 47 participants were excluded. Finally, based on univariate outlier analyses based on psychological burden scores another two outliers were excluded. The multivariate outlier analyses did not reveal any more outliers. In this sample, Cronbach’s alfa for the PFC measure was .745, for the sexual function measure .968, and the psychological burden measure .946.

**Table 3.** Prevalence rates of pelvic floor complaints in the full sample, and descriptive statistics of pelvic floor complaint severity, sexual functioning, and psychological burden in participants receiving and not receiving pelvic physical therapy.

	Total sample	No pelvic physical therapy		Pelvic physical therapy		Between- group differences
	PFC prevalence %	Mean severity	SD	Mean severity	SD	*F*
**Pelvic floor** ** complaints**	**93**	** 3.37**	** 2.66**	** 5.24**	** 2.70**	18.97***
Urinary incontinence	41	0.38	0.62	0.55	0.76	3.47
Fecal incontinence	9	0.06	0.22	0.10	0.39	1.03
Micturition problems	53	0.43	0.62	0.68	0.73	14.08***
Defecation problems	55	0.49	0.65	0.60	0.72	4.61*
Pelvic organ prolapses	9	0.13	0.46	0.13	0.49	0.02
Pelvic pain	44	0.52	0.83	1.55	1.10	125.71***
Painful intercourse	65	1.37	1.30	1.62	1.27	3.40
**Sexual** **functioning**		**15.69**	**6.30**	**14.77**	**6.65**	** 1.42**
Desire		3.26	1.20	3.17	1.09	0.70
Arousal		3.95	1.84	3.59	1.96	4.94*
Lubrication		4.45	2.04	4.16	2.26	2.51
Orgasm		4.03	2.00	3.85	2.14	0.53
**Psychological** ** burden** ^a^		**12.96**	**14.24**	**23.33**	**17.50**	45.28***

Note: N=542. SD=Standard deviation. The overall scores of pelvic floor complaints, sexual functioning, and psychological burden are indicated in bold. N=483 in the F tests. ^a^ Pelvic Floor Complaint-related Psychological Burden.

The differences in severity of the different types of PFC and the different subscale scores of sexual functioning between women who do and do not receive help were analyzed using MANOVA. Significant differences were found with regard to PFC severity, *F*=18.97, *p*<0.001. More specifically, women who did and did not receive help differed with regard to the severity of micturition problems, *F*=14.08, *p*<0.001, defecation problems, *F*=4.61, *p*=0.032, and pelvic pain, *F*=125.71, *p*<0.001. No significant differences in sexual functioning were found, despite the significant difference in sexual arousal between the two groups of participants, *F*=4.94, *p*=0.027. Between-group differences in psychological burden were tested using ANOVA. A significant difference in the level of psychological burden was found, *F*=45.28, *p*<0.001.

The main research question concerned the predictive value of pregnancy, parity, PFC severity, level of sexual functioning, and women’s psychological burden for receiving help. Before further analyses, PFC severity, sexual functioning, and psychological burden scores were standardized. The significant omnibus test (χ ^2^=127.22, df=14, *p*<0.001), and the non-significant Hosmer and Lemeshow tests (χ ^2^=10.27, df=8, *p*=0.247) indicated a good model fit with the data. Pregnancy (B=2.562, β=12.956, *p*<0.001), parity (B=1.558, β=4.748, *p*<0.001), PFC severity (B=0.595, β=1.812, *p*=0.018), and psychological burden (B=0.817, β=2.264, *p*<0.001) were significant predictors of receiving help. The odds that a pregnant woman receives help are 12.96 times higher than that of nulliparous women. The odds that a parous woman receives help are 4.75 times higher than that of nulliparous women. An increase of one unit of PFC severity increased the odds of receiving help 1.81 times. An increase of one unit of psychological burden increased the odds of receiving help 2.26 times. Sexual functioning did not predict receiving help.

A significant and negative interaction effect was found between PFC severity and psychological burden (B=-0.425, β=0.654, *p*=0.003). This outcome indicated that PFC severity moderated the prediction of help-seeking by psychological burden. Predicting the likelihood of help-seeking by the level of psychological burden was better at lower levels of PFC severity. Pregnancy and parity significantly increased the likelihood of receiving help, but their interaction effects with PFC severity, sexual functioning, or psychological burden were not significant. In addition, no significant interaction effects were found between PFC severity and sexual functioning, nor between sexual functioning and psychological burden (see Table 4).

**Table 4.** The predictive value of pregnancy, parity, pelvic floor complaints, sexual functioning, and psychological burden for receiving help in pelvic physical therapy practice.

Predictor variable	B	95%CI		SE	β
		LL	UL		
**Pregnant (Indicator)**	** 2.562**	**5.705**	**29.421**	**0.418**	12.956 ***
**Parous (Indicator)**	** 1.558**	**2.516**	** 8.960**	**0.324**	4.748 ***
**Severity of pelvic floor ** **complaints (PFC)**	** 0.595**	**1.106**	** 2.968**	**0.252**	1.812 *
Sexual functioning (SF)	0.246	0.756	2.164	0.268	1.279
**Psychological burden** ^a^ **(PB)**	** 0.817**	**1.462**	** 3.507**	**0.223**	2.264 ***
Pregnant *PFC	0.066	0.368	3.102	0.544	1.068
Pregnant *SF	0.115	0.465	2.707	0.449	1.112
Pregnant *PB	-0.340	0.262	1.934	0.510	0.711
Parous *PFC	0.278	0.703	2.480	0.321	1.321
Parous *SF	-0.014	0.560	1.734	0.288	0.986
Parous *PB	-0.331	0.405	1.274	0.292	0.718
SF *PFC	0.075	0.679	1.266	0.159	0.927
PB *PFC	**-0.425**	**0.495**	** 0.864**	**0.142**	0.654 **
SF *PB	-0.048	0.746	1.218	0.125	0.953

Note: *N* = 483. * *p* < .05, ** *p* < .01, *** *p <* .001. PFC=Pelvic floor complaints, SF=Sexual functioning, PB=Psychological burden. ^a^ Pelvic Floor Complaint-related Psychological Burden.

## Discussion

This study explored whether pregnancy, parity, PFC severity, level of sexual functioning, and psychological burden predicted if women suffering from PFC received help in PPT practice. Conspicuously, against expectations, 93% of all participants were found to be experiencing one or more PFC, implying that several of the women who were included in the healthy sample also reported one or more PFC. Of these women with PFC, only 24% received help in PPT practice. PFC severity was negatively associated with sexual functioning and positively with the psychological burden. Pregnancy and parity were found to be significant predictors of receiving help. These particular circumstances in women’s lives apparently increase the likelihood of receiving help when experiencing PFC.

In the total sample, all correlations between PFC severity, sexual functioning, and psychological burden were significant. However, variations in the significance of correlations emerged in pregnant, parous, and nulliparous women. The correlations between PFC severity and sexual functioning were found not significant in nulliparous women with PPT. Furthermore, the correlations between sexual functioning and psychological burden were not significant in pregnant and parous women with and without PPT. These outcomes show a relevant overlap with previous results in our lab ^54^, indicating that pregnant and particularly parous women mainly experience sexual functioning problems as a result of pregnancy and childbirth-related PFC. This may explain why their level of sexual functioning and psychological burden in this study were significantly correlated with PFC severity. In nulliparous women who receive help, PFC severity and sexual functioning problems were significantly associated with psychological burden, which aligns with the previous results on sexual function problems and distress about relationships and sexual performance ^54^.

PFC severity was, as was expected, a significant predictor of receiving help. A conspicuous finding was that 69% of the participants with PFC in this study did not receive help. Participants may have appraised their PFC as not severe enough, as part of the aging process, or as insoluble, and may have adapted their behavior to deal with their PFC or simply accepted their presence ^12, 40^. The different ways of dealing with PFC severity could be further explored in future research. In addition, it might be relevant to investigate which specific types of PFCs are accompanied by a high psychological burden.

Women’s psychological burden was found to be a significant, and stronger statistical predictor for receiving help than PFC severity. The higher psychological burden in women with PFC who did receive help indicates that this burden may have been an important additional reason for receiving help. This finding warrants further exploration in prospective research.

Essentially, the content of women’s psychological burden mainly consists of feeling angry, wronged, and helpless, and of loss of control in the context of their daily, social, and sexual functioning, and in their intimate relationships ^48^. These types of distress related to present PFC also predict whether or not women receive help. This clarification of women’s psychological burden with PFC provides opportunities to better address and evaluate it in clinical practice.

In addition, the significant negative interaction effect of PFC severity and psychological burden indicates that psychological burden is a stronger predictor of receiving help at lower compared to higher levels of PFC severity. When PFC severity exceeds a certain—yet undefined—level, women’s psychological burden appears to become less relevant in predicting help. There might be a cut-off point in PFC severity beyond which the expected effectiveness of PPT treatment declines or disappears. These findings might imply the existence of a certain range of PFC severity in which women expect PPT to be effective, and in which PPT could be regarded as most effective. When PFC severity is very high, women might feel that they require help from specialists, such as gynecologists, urologists, proctologists, sexologists, or psychologists rather than from pelvic physical therapists, regardless of the accompanying psychological burden. Therefore, the present findings indicate the necessity to further investigate this area of pelvic healthcare. More insight is required into whether, and to what extent, women’s psychological burden needs addressing in PPT practice, given that adequate treatment could also reduce women's psychological burden without specifically addressing this burden. Furthermore, our findings prompt the further examination of psychological burden as an indicator during the screening process to find the most appropriate healthcare provider. This knowledge may help, for instance, general practitioners when screening women for adequate referrals to pelvic healthcare.

The psychological burden should also be further examined as an outcome measure in PPT practice. More information on this topic may reveal a necessity to include the psychological burden in PPT screening and intake and help to identify a cut-off point beyond which one could better refer to other pelvic healthcare providers. The PFC-PBI can be a helpful instrument in these contexts ^48^. More knowledge about the psychological burden of PFC could—ultimately—improve pelvic healthcare for women, and stimulate better collaboration between pelvic healthcare providers.

Against expectations, women’s level of sexual functioning was not predictive of receiving help. An explanation could be that women think that PPT practice is not the most appropriate place to receive help for sexual functioning problems. The absence of significant differences in sexual functioning between women who did and did not receive help may also explain why sexual functioning is not predictive of receiving help in PPT practice. The significant correlations between PFC severity and sexual functioning in the total sample suggest a strong negative influence of PFC severity on sexual functioning. This association, however, appears not strong enough to make these variables and their interaction significantly predictive of receiving help. An additional speculative explanation for this might be that previous research did not show a causal relationship between PFC and sexual functioning but suggested the influence of other factors, such as quality of life and sexual pleasure causing women’s sexual functioning problems ^55^.

This complexity is further emphasized by the findings in this study that in the total sample and among nulliparous women who received help, in contrast to pregnant and parous women, significant associations existed between their level of sexual functioning and psychological burden. It might be that, for example, the combination of painful intercourse and distress about being a capable sexual partner or becoming pregnant is so problematic for nulliparous women that they turn to PPT in an attempt to solve their sexual distress. However, the interaction between sexual functioning and women’s psychological burden was also not a significant predictor of receiving help. These outcomes only yield many additional questions that require further research on sexual functioning problems in relation to PFC and its treatment.

### Limitations

The first limitation of this study pertains to the inventory of PFC severity. Participants answered questions about the presence and severity based on various common symptoms of the seven PFC. Factors, such as daily and social activities related to lifestyle and personal circumstances, diet, fluid intake, toilet behavior, sleep quality, family, the type of work, and the nature of sports that affect PFC were not inventoried in this survey. This was purposely done to avoid overloading participants. In future research, the inventory of PFC could be optimized by primarily offering women questions about their self-perceived presence of PFC before asking questions about severity. In addition, questions about lifestyle and adaptive behavior when not receiving help for PFC could be added. An important potential limitation is that there may have been women with PFC who wanted help in PPT practice, but who never received help because of financial reasons, not knowing of the possibility to receive help, or because they received help from other healthcare providers. This should be addressed in future research on this topic.

A third limitation pertains to recruitment issues. Pregnant, parous, and nulliparous women were not equally represented, nor were women with and without PFC. The differences in the number of participants in the subgroups may have influenced outcomes. The group of pregnant women was relatively small. Furthermore, it proved difficult to reach and motivate women without PFC to participate. Reasons for not participating could have been uneasiness with the survey topic, lack of time, distraction and forgetting to register for or complete the survey, or labeling the study as not applicable to oneself. The option to fill out the survey in stages resulted in missing data on later tasks in the survey. System-induced automatic reminders were installed during the course of the data collection period to prompt women to finish the various tasks in the survey after initiation. Earlier installation might have prevented or reduced the initial substantial amount of missing data.

## Conclusion

In conclusion, PFC severity, women’s psychological burden, and their interaction were found significant predictors of receiving help in PPT practice. Psychological burden is a stronger predictor of help-seeking at lower compared to higher levels of PFC severity. Outcomes suggest that above a certain level of PFC severity, women’s psychological burden becomes less relevant in predicting receiving help. Pregnancy and parity also predicted help-seeking behavior. Against expectations, sexual functioning was not found predictive of receiving help, despite the fact that sexual functioning is negatively associated with PFC severity. The sample may not be representative of all women with PFC in the Netherlands and other countries, and, therefore, the results are not guaranteed to be generalizable. Further research is needed to unravel the full implications of these findings.

## Ethics and consent

The study protocol was approved by the Ethical Review Board of the Open University of the Netherlands on May 29th, 2019/No. U2019/03973/HVM. Participants gave informed written consent to participate and were assured of anonymous use and publication of the data they provided.

## Data Availability

OSF: Predictors to seeking help in Pelvic Physical Therapy Practice. https://doi.org/10.17605/OSF.IO/Z6W7S ^56^. This project contains the following underlying data: Final Original File JVV Study.sav (Raw data file) OSF: Predictors to seeking help in Pelvic Physical Therapy Practice. https://doi.org/10.17605/OSF.IO/Z6W7S ^56^. JVV DATA FINAL SYNTAX.sps (SPSS syntax file) 4DSQ English.pdf FSFI Scoring.pdf pelvic-floor-distress-index.pdf Questionnaire NL-EN.docx Data are available under the terms of the Creative Commons Attribution 4.0 International license (CC-BY 4.0).
