# Supplementary material for: Sexual functioning is not, but psychological burden is predictive for receiving help in pelvic physical therapy practice: A cross-sectional study
Source: Open Res Eur. 2024 May 20;3:141. Originally published 2023 Sep 7. [Version 2] doi: 10.12688/openreseurope.16138.2 (PMC11143402; doi:10.12688/openreseurope.16138.2)
Supplement: Peer review history

# Reviewer report: Reviewer response for version 2

**DOI:** 10.21956/openreseurope.19121.r40668

**Published:** 2024-05-31

**Authors:** Erika L. Kelley

**Affiliations:** 1 UH Cleveland Medical Center, Cleveland, OH, USA; 2 Departments of Obstetrics and Gynecology and Urology, University Hospitals Cleveland Medical Center, Cleveland, OH, USA

The authors were responsive to reviewer suggestions and questions. The revised text is a well-written article presenting findings from a study identifying correlates of receiving help among a sample of women in a physical therapy practice. Results of this study may inform the management of pelvic floor complaints among women and seem to reiterate the importance of interdisciplinary care. I have no further comments.

If applicable, is the statistical analysis and its interpretation appropriate?

Yes

Is the study design appropriate and is the work technically sound?

Yes

Is the work clearly and accurately presented and does it engage with the current literature?

Yes

Are the conclusions drawn adequately supported by the results?

Partly

Are sufficient details of methods and analysis provided to allow replication by others?

Yes

Are all the source data and materials underlying the results available?

Yes

Reviewer Expertise:

Female sexual dysfunction; mental health; health psychology

I confirm that I have read this submission and believe that I have an appropriate level of expertise to confirm that it is of an acceptable scientific standard.

# Reviewer report: Reviewer response for version 1

**DOI:** 10.21956/openreseurope.17421.r39201

**Published:** 2024-04-19

**Authors:** Laís Campos de Oliveira

**Affiliations:** 1 Universidade Estadual do Norte do Paraná, Paraná, Brazil

Congratulations to the authors.

- Well-written introduction details the objective of the study and justifies its carrying out due to the high prevalence of women with complaints related to the pelvic floor.
- I suggest adding a brief explanation of the benefits of pelvic physiotherapy in these cases, in order to justify the need to seek it.
- The terminology “help-seeking behaviors” did not seem to me to reflect very well whether or not pelvic physiotherapy was performed. I suggest replacing it with terms that best suit it.
- Characteristics of the included volunteers are well described.
- Possible reasons for bias are stated in the text and have been minimized where possible.
- The outcome measures sought were well described in terms of their scoring and interpretation of the results.
- Strong point: Sample calculation was carried out and different forms of recruitment were sought, covering a larger and more diverse population.
- The statistical analysis was well described.
- In some cases throughout the text, variations of the term “predictor” make it difficult to understand the actual methodology of the study, which is to correlate pelvic floor complaints, sexual function and psychological burden with the search or not for physiotherapeutic treatment.
- Well-written discussion and provides probable explanations for each result found.
- Well-described methodology and following high quality criteria.
- The results of this study highlight the importance of biopsychosocial assessment by physiotherapy professionals.
- This study will contribute to science for a better interpretation of this topic that is so important for society.

If applicable, is the statistical analysis and its interpretation appropriate?

Yes

Is the study design appropriate and is the work technically sound?

Yes

Is the work clearly and accurately presented and does it engage with the current literature?

Yes

Are the conclusions drawn adequately supported by the results?

Yes

Are sufficient details of methods and analysis provided to allow replication by others?

Yes

Are all the source data and materials underlying the results available?

Yes

Reviewer Expertise:

NA

I confirm that I have read this submission and believe that I have an appropriate level of expertise to confirm that it is of an acceptable scientific standard.

# Response

**Published:** 2024-04-23

**Authors:** Alma Brand

**Affiliations:** Psychology, Open Universiteit, Heerlen, Limburg, The Netherlands

We would like to thank reviewer Laís Campos de Oliveira for the compliments and recommendations. **Our responses are written in italics under each point of feedback.** Well-written introduction details the objective of the study and justifies its carrying out due to the high prevalence of women with complaints related to the pelvic floor.           

*Thank you.*

 I suggest adding a brief explanation of the benefits of pelvic physiotherapy in these cases, in order to justify the need to seek it.

*A brief explanation has been added. (in the hope of improving their pelvic or sexual health)*

 The terminology “help-seeking behaviors” did not seem to me to reflect very well whether or not pelvic physiotherapy was performed. I suggest replacing it with terms that best suit it.

*This comment was also made by the other reviewer* *, so we respond with the same text: We have reframed the terminology to receipt of pelvic physical therapy, rather than seeking help. Thank you for pointing this out, we fully agree this clarifies the content of the paper.*

 Characteristics of the included volunteers are well described.            

*Thank you.*

 Possible reasons for bias are stated in the text and have been minimized where possible. *Thank you.*

 The outcome measures sought were well described in terms of their scoring and interpretation of the results.            

*Thank you.*

 Strong point: Sample calculation was carried out and different forms of recruitment were sought, covering a larger and more diverse population.            

*Thank you for this compliment.*

 The statistical analysis was well described.            

*Thank you.*

 In some cases, throughout the text, variations of the term “predictor” make it difficult to understand the actual methodology of the study, which is to correlate pelvic floor complaints, sexual function, and psychological burden with the search or not for physiotherapeutic treatment.

*Thank you for this feedback.*

*We received a similar comment from the other reviewer, and therefore, we will respond with the same text:*

*We use statistical prediction, rather than clinical prediction. Given this perspective, the use of a predictor, rather than a correlate or similar is preferred, to avoid misinterpretation of the results. There is no intention to determine causality, we are looking at the prediction of group membership (receiving or not receiving therapy). Therefore, no textual adaptations of this terminology were made.*

 Well-written discussion and provides probable explanations for each result found. *Thank you.*

 Well-described methodology and following high-quality criteria.            

*Thank you.*

 The results of this study highlight the importance of biopsychosocial assessment by physiotherapy professionals.            

*We agree!*

 This study will contribute to science for a better interpretation of this topic that is so important for society.            

*Thank you.*

# Reviewer report: Reviewer response for version 1

**DOI:** 10.21956/openreseurope.17421.r35609

**Published:** 2023-11-10

**Authors:** Erika L. Kelley

**Affiliations:** 1 UH Cleveland Medical Center, Cleveland, OH, USA; 2 Departments of Obstetrics and Gynecology and Urology, University Hospitals Cleveland Medical Center, Cleveland, OH, USA

This paper describes results from a survey study aimed at identifying predictors of help-seeking for pelvic floor therapy.  Results of this study have implications for the identification of the needs of women with pelvic floor complaints. The methodology and results are clearly described. Strengths of this study include the use of power analysis to determine adequate sample size and the attempts to recruit a diverse sample in terms of parity and experience of PFC. The description of the statistical analysis is very clear.  Results indicate that psychological burden was an important factor in receiving PFT and may indicate the importance of screening for psychological burden and distress levels. Results also appear to indicate that nulliparous women may be an underserved population with respect to PFT.

 My comments and recommendations are as follows:

 The authors describe the importance of understanding help-seeking behaviors for women with pelvic floor complaints. However, it appears the methodology may better reflect the construct of receipt of PFT (i.e., women receiving PFT vs. those not receiving PFT), rather than help-seeking behavior and it may be helpful to reframe the terminology in this way.  For example, the sample includes women who were and were not actively in PFT, which is not always equivalent to help-seeking. It is possible that some women sought or would want to seek help, but experienced barriers to receipt of PFT.  Some research identifies the underassessment and under-treatment of sexual and pelvic concerns in women, as well as minimization of pain.  Relatedly, it may help readers understand the context and possible generalizability of results if there is a description of the assessment and referral practices for PFC typical in the setting where the study occurred. Perhaps there are fewer such access barriers experienced in this sample.

 With the current methodology, it is not possible to determine causal effect and it may be important to temper language away from “predictor” to “correlate” or similar. A related limitation is that it is possible that for the subsample who are in PFT, their PFC severity ratings are lower because they have improved from being in PFT.  This is reflective of the limited ability to determine causality with the present design. This study provides an important first step in identifying possible contributing factors for engagement in PFT. Future longitudinal work building on this project may be helpful to better understand predictors of help-seeking behaviors for PFT.  In future research, it may also be helpful to examine partner-related variables given increasing studies showing the importance of partner relationship factors in women’s experience with sexual/pelvic pain.

 It appears that individuals who were not sexually active were included in the FSFI assessment, and that ‘did not try having intercourse’ was scored as highest severity of PFC. Although the authors note the full FSFI score was not used, there is research indicating that the validity of the FSFI is compromised when non-sexually active individuals are included in the scoring (e.g., Meston et al., Journal of Sexual Medicine, 2019). For example, there may be a number of other reasons an individual did not attempt intercourse rather than pelvic floor complaints.  To this end, it may be interesting and helpful to conduct a sub-analysis of the samples of sexually active vs. non-sexually active individuals.

 The authors note the limitation in the PFC measure. If possible, it would be helpful to know the internal consistency reliability (Cronbach’s alpha) for this sample.  Future research may consider evaluation of the psychometric properties of the scale developed for this study (e.g., for use with a longitudinal design) that could be used in future longitudinal work.

 A minor comment: The first sentence of the third paragraph in the Discussion section ("PFC severity was, as was expected, ...") seems inconsistent with the fourth sentence of paragraph 1 of Discussion ("despite the significant between-group differences in PFC severity, it did not predict seeking help"). I read the latter as meaning PFC did not predict help-seeking. Clarification might be helpful.

 I appreciate the opportunity to review this manuscript that highlights the correlates of women’s engagement in PFT.

If applicable, is the statistical analysis and its interpretation appropriate?

Yes

Is the study design appropriate and is the work technically sound?

Yes

Is the work clearly and accurately presented and does it engage with the current literature?

Yes

Are the conclusions drawn adequately supported by the results?

Partly

Are sufficient details of methods and analysis provided to allow replication by others?

Yes

Are all the source data and materials underlying the results available?

Yes

Reviewer Expertise:

Female sexual dysfunction; mental health; health psychology

I confirm that I have read this submission and believe that I have an appropriate level of expertise to confirm that it is of an acceptable scientific standard, however I have significant reservations, as outlined above.

## References

[1] : Scoring and Interpretation of the FSFI: What can be Learned From 20 Years of use?. *J Sex Med* .2020;17(1) : 10.1016/j.jsxm.2019.10.007 17-25 10.1016/j.jsxm.2019.10.007 31735616

# Response

**Published:** 2024-04-23

**Authors:** Alma Brand

**Affiliations:** Psychology, Open Universiteit, Heerlen, Limburg, The Netherlands

We would like to thank reviewer Erika L. Kelley for her kind and constructive feedback and recommendations.  

**Response to the first recommendation concerning seeking help vs. receiving help:** We have reframed the terminology to receipt of pelvic physical therapy, rather than seeking help. Thank you for pointing this out, we fully agree this clarifies the content of the paper.  

**Response to the second recommendation concerning the suggestion to use correlate or similar rather than predictor:** We use statistical prediction, rather than clinical prediction. Given this perspective, the use of a predictor, rather than a correlate or similar is preferred, to avoid misinterpretation of the results. There is no intention to determine causality, we are looking at the prediction of group membership (receiving or not receiving therapy). Therefore, no textual adaptations of this terminology were made.  

**Response to the third recommendation concerning sexually active vs. non-sexually active women:** This is very sharply noticed, thank you for pointing this out to us. The part of the sentence about sexual activity before filling out the FSFI is a textual error that has escaped our notion, and, therefore, is redundant because being sexually active or not is already integrated into the FSFI. We did include the question about negative sexual experiences because this may affect women’s level of sexual functioning. An explanatory sentence has been added to the text in the paragraph in the method section, and in the result section, which indicates that there was no significant difference in the level of sexual functioning between women with and without negative sexual experiences.  

**Response to the fourth recommendation concerning the internal consistency of the PFC measure:** We have provided Cronbach’s Alfa for the seven pelvic floor complaints as calculated and used in our sample.  

**Response to the fifth recommendation concerning an inconsistency in the text of the third paragraph of the discussion:** Thank you for pointing this second textual error out to us. The fourth sentence of the first paragraph of the discussion is incorrect and, therefore, deleted.
